# Hyperkinetic Movement Disorder in *KARS1*-Related Disease: An Illustrative Video-Recorded Case and Narrative Literature Review

**DOI:** 10.3390/neurolint17090143

**Published:** 2025-09-07

**Authors:** Veronica Ferasin, Arianna Raicich, Caterina Ancora, Ilaria Bonemazzi, Alessandro Di Paola, Ignazio D’Errico, Margherita Nosadini, Claudio Ancona, Maria Federica Pelizza, Matteo Cassina, Irene Toldo

**Affiliations:** 1 Child Neurology and Neurophysiology Unit, Department of Women’s and Children’s Health, Padua Hospital, University of Padua, 35128 Padova, Italy; 2 Child Neuropsychiatry Unit, San Bortolo Hospital, ULSS 8 Berica, Vicenza, 36100 Vicenza, Italy; 3 Neuroradiology Unit, Department of Neurosciences, Padua Hospital, University of Padua, 35128 Padova, Italy; 4 Clinical Genetics Unit, Department of Woman’s and Child’s Health, Padua Hospital, University of Padua, 35128 Padova, Italy

**Keywords:** *KARS1*, hyperkinetic movement disorder, CNS calcifications, brain atrophy, LEPID

## Abstract

Background: Aminoacyl-tRNA synthetases (ARSs) are a group of enzymes responsible for the first step of protein translation. Among them, the KARS1 gene encodes lysyl-tRNA synthetase 1, an enzyme essential for charging tRNA-Lys with lysine in both the cytoplasm and mitochondria. Mutations in KARS1 are associated with a wide range of clinical phenotypes, including leukoencephalopathy, hereditary deafness, peripheral neuropathies, and multisystemic involvement. Methods: We hereby report a detailed case study of a 15-month-old boy presenting at age 5 months with developmental delay, microcephaly, hypotonia, sensorineural deafness, retinopathy, visual impairment, nystagmoid eye movements, and hepatic and immuno-hematological abnormalities. In addition, he exhibited a severe hyperkinetic movement disorder, not previously reported in the literature, and developed epilepsy at 13 months. Genetic testing identified two rare compound heterozygous variants in the KARS1 gene. Results: With this report, we aim to contribute to the expanding of both the clinical phenotype and the allelic spectrum of lysyl-tRNA synthetase-related disorders. Our study also includes a review of previously described KARS1 cases presenting with movement disorders. Conclusions: Our findings further highlight the importance of assessing systemic involvement and performing brain and spinal neuroimaging, as well as implementing genetic screening, in infants presenting with global developmental delay, sensory deficits, and movement disorders—features that may suggest a mitochondrial disorder such as those involving ARS mutations.

## 1. Introduction

*KARS1*-related syndrome is a rare autosomal recessive disorder caused by biallelic pathogenic variants in the *KARS1* gene [1]. The *KARS1* gene maps to chromosome 16q23.1 [1] and encodes for both the mitochondrial and cytoplasmic forms of lysine t-RNA synthetase 1 [2].

Homozygous and compound heterozygous variants in *KARS1* have been linked to different diseases with neurologic and systemic (hepatic, cardiological, and immuno-hematologic) involvement [2,3,4,5,6,7,8,9,10].

The spectrum of neurological phenotypes is broad, encompassing hereditary deafness, Charcot–Marie–Tooth neuropathies, progressive leukoencephalopathies with infant or adult onset, and mitochondrial hepato-encephalopathy with microcephaly and leopard-like retinopathy [1,2,11,12]. Epilepsy and movement disorders have also been reported but have not been extensively described to date.

In this report, we describe a novel case of early infantile *KARS1*-related syndrome, presenting with a prominent dyskinetic choreo-ballistic movement disorder, along with progressive multiorgan involvement.

The aim of our study is to highlight novel key clinical features that may be crucial in the diagnosis of *KARS1*-related syndromes, thereby expanding the phenotypic spectrum and contributing to a more detailed characterization of the disease.

## 2. Methods

In this report, we describe a novel patient with early infantile *KARS1*-related syndrome.

Written informed consent for the publication of the case study was obtained from caregivers. The study was conducted in accordance with the ethical principles of the Declaration of Helsinki and its subsequent amendments.

Furthermore, we conducted a narrative review via PubMed and Medline (up to June 2025) of the previously described cases of *KARS1*-related disorders, employing the following advanced search strategy: “KARS” OR “*KARS1*” OR “lysyl-tRNA synthetase” OR “lysyl-tRNA synthetase 1” AND (mutation OR mutations OR mutated OR variant OR variants OR deficiency OR “related disease” OR “related disorder” OR syndrome). Studies published in languages other than English were excluded.

We then performed a manual reference check of the retrieved articles in order to include those describing *KARS1*-related disease presenting with movement disorders.

## 3. Case Description

### 3.1. Medical History

We present the case of a second-born male, delivered at 40 + 6 gestational weeks to healthy, non-consanguineous parents. Pregnancy was complicated by oligohydramnios, intrauterine growth restriction, and prenatal detection of choroid plexus cysts documented by ultrasound and fetal MRI. A prenatal array CGH, performed on chorionic villi, was negative.

At birth, the patient presented with relative microcephaly, with a head circumference of 33 cm (9th percentile CDC), a length of 50 cm (50th percentile CDC), and a weight of 3010 g (18th percentile CDC).

Hearing screening with the Otoacoustic Emission Tone Test revealed abnormal results in the right ear and borderline results in the left ear. At 2 months of age, auditory brainstem response testing showed elevated auditory thresholds and absent otoacoustic emissions, suggesting a bilateral severe hearing loss.

The patient was referred to our hospital at age 5 months, due to psychomotor developmental delay and failure to thrive (with height and weight falling below the 3rd percentile).

Physical examination did not reveal significant abnormalities except for bilateral clinodactyly of the fifth toe.

Neurological evaluation revealed an alert and responsive infant, with absent visual fixation, tracking, and social smile. The boy had severe cervical–axial hypotonia and poor spontaneous antigravity movements. Deep tendon reflexes were brisk and symmetric. Horizontal nystagmus bursts and jerky eye movements were present (Video S1, Supplementary Materials). In addition, he displayed a subcontinuous hyperkinetic movement disorder (Video S2, Supplementary Materials), associated with orolingual dystonia, grimacing, and tongue protrusions (Video S3, Supplementary Materials).

### 3.2. Results of Imaging Data

Cranial ultrasound at 2 months showed dilatation of the lateral ventricles bilaterally.

**Figure 1:**
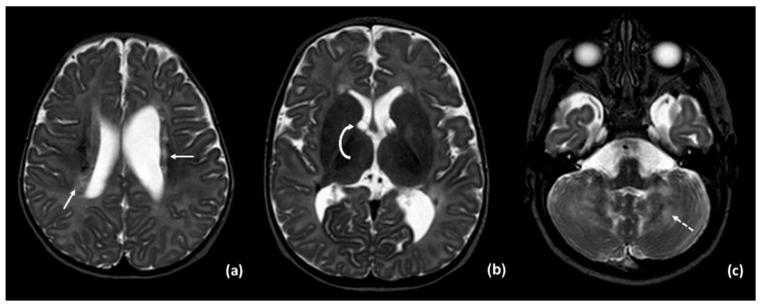
MRI T2-weighted images show: (**a**) foci of signal alterations in the periventricular white matter (solid arrow); (**b**) small subependymal cysts (curved arrow); (**c**) signal abnormalities in the cerebellum (dashed arrow).

**Figure 2:**
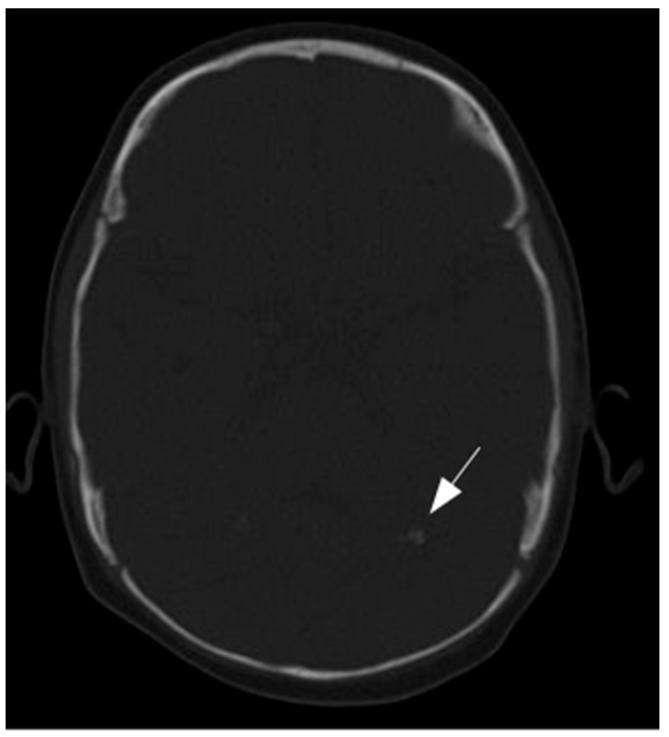
CT scan shows mild calcifications of the cerebellum (solid arrow).

Brain MRI performed at 5 months of age revealed mildly dysmorphic and asymmetric lateral ventricles with subependymal cysts located near the foramina of Monro and signal abnormalities involving the cerebellum and periventricular white matter (Figure 1). A head CT scan, performed to investigate the cause of hearing loss, revealed cerebellar calcifications but was otherwise unremarkable, showing no identifiable cause of the deafness (Figure 2).

**Figure 3:**
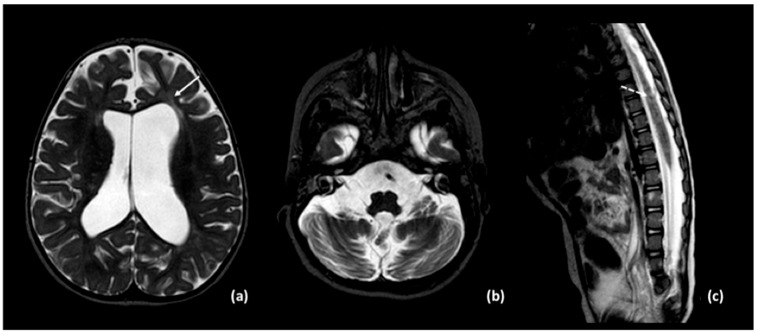
MRI T2-weighted images show: (**a**) ventriculomegaly due to atrophy and mild alteration of periventricular white matter (solid arrow); (**b**) severe cerebellar atrophy; (**c**) multiple mild T2 hypointense foci within the spinal cord, suggestive of calcifications (dashed arrow).

**Figure 4:**
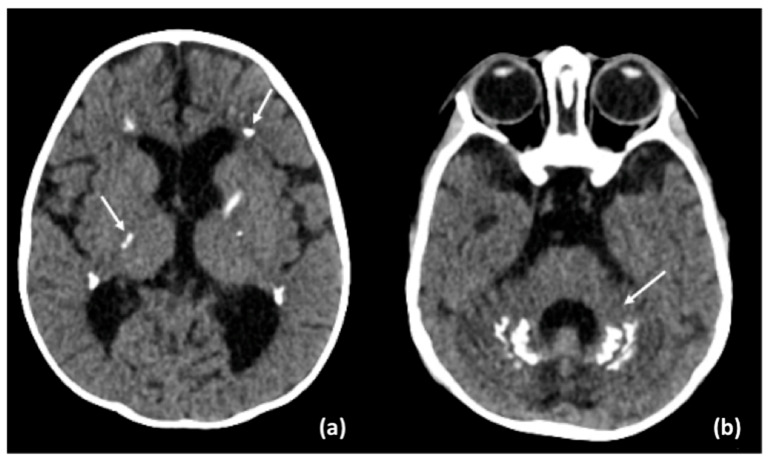
CT scan shows: (**a**) calcifications of periventricular white matter and internal capsules (solid arrows); (**b**) cerebellar calcifications (solid arrow).

Brain MRI and CT performed at 15 months showed severe atrophic progression, predominantly involving the cerebellum, with dilation of the ventricular system and extensive and increased calcifications of the periventricular white matter, internal capsules, and cerebellum (Figure 3 and Figure 4). Mild alteration of the periventricular white matter was observed. Spinal cord MRI revealed diffuse signal alterations suggestive of calcifications (Figure 3).

**Figure 5:**
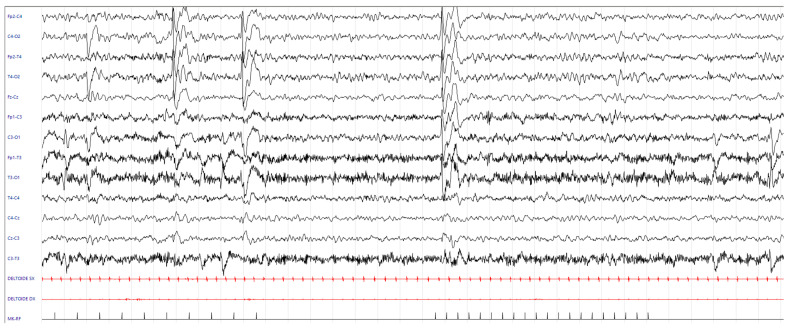
EEG during wakefulness (age 5 months): epileptiform abnormalities involving the right fronto-centro-temporal and centro-temporo-occipital areas and spreading to adjacent and contralateral regions, increasing during IPS. EEG parameters: 15 mm/s; sensitivity: 14 uV/mm; bandpass filter: 1–70 Hz; notch on. The X-axis represents time, with 1 second between each vertical grey line. The horizontal black traces correspond to the scalp EEG, the horizontal red traces correspond to the polygraphic recording of the right and left deltoid muscles, and the short vertical black lines correspond to the light stimuli of the IPS.

Longitudinal EEG studies (since age 5 months) revealed frequent epileptiform abnormalities in the bilateral posterior regions, either synchronous or asynchronous, which increased during sleep and were enhanced by intermittent photic stimulation (IPS) (Figure 5).

**Figure 6:**
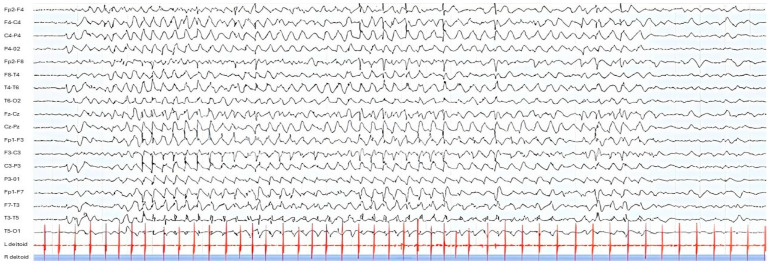
Ictal EEG pattern during sleep. EEG parameters: 15 mm/s; sensitivity: 50 uV/mm; notch on. The X-axis represents time, with 1 second between each vertical grey line. The horizontal black traces correspond to the scalp EEG, the horizontal red traces correspond to the polygraphic recording of the right and left deltoid muscles and the blue bar represents the sleep stage.

Since 7 months of age, EEG abnormalities were prevalent during wakefulness, involving the right fronto-centro-temporal and centro-temporo-occipital areas and spreading to adjacent and contralateral regions; IPS continued to exacerbate these abnormalities. An EEG performed at 13 months of age revealed numerous atypical absence-type EEG patterns elicited by IPS. Treatment with ethosuximide was thus initiated, leading to a beneficial response, with a reduction in these EEG patterns. At age 15 months, he presented with frequent (multiple daily) brief focal motor seizures, lasting 20–30 s, characterized by left version of head and eyes, subtle bilateral brow elevations, and tonic–clonic movements of the arms with a tonic asymmetric posture suggesting the “4 sign figure” with an extended left arm. An EEG at age 15 months displayed, during wakefulness and sleep, bursts of epileptiform discharges, prevalent in the bilateral posterior regions, exacerbated by IPS. An ictal EEG pattern was recorded showing, during wakefulness, clonic involvement of the upper limbs, and during sleep, a focal tonic component (Figure 6). Add-on therapy with levetiracetam was then started. Antiseizure medications were also selected with consideration of the patient’s hepatic and hematological comorbidities.

Oculomotor function (age 5 months) was characterized by esophoria and erratic eye movements, with predominantly horizontal nystagmus bursts with a pendular component associated with head movements; visual fixation and tracking were absent. Fundus examination showed pigmentary mottling at the posterior pole and mid-peripheral retina. At 7 months, visual acuity was limited to light perception.

Visual evoked potentials (VEPs) were performed at 5 months of age, showing normal cortical activation and signal transmission in response to flash stimulation in both eyes. At 7 months of age, VEPs were present but reduced in amplitude bilaterally. The examination was repeated at 15 months of age; however, the procedure was interrupted due to seizure provocation.

An ophthalmologic evaluation at last follow-up (15 months) revealed severe visual inattention, with a preferred gaze position directed upward, light gazing, occasional fixation and tracking of light, nystagmus with fine and broad jerks, and a visual acuity with Teller Acuity Cards of 1.3 cycles at 20 cm (equal to approximately 1/60) not further improved in the preferred gaze position.

Systemic involvement was also documented since age 5 months, including hepatic and hematologic abnormalities. Abdominal ultrasound at age 5 months showed a heterogeneous hyperechoic echotexture with multiple oval hypoechoic lesions (largest 12 mm), an apparently normal portal vein, and a spleen at the upper limit of normal size (64 mm). Subsequent evaluations revealed worsening of portal flow (reduced to 10 cm/s) and increased spleen size (75 mm).

Abdominal MRI (age 5 months) showed a morphologically normal liver with diffusely heterogeneous signal intensity, periportal thickening, and hyperintensity and ill-defined nodular areas, the most prominent in segment 7 (7 mm), presenting intense arterial phase enhancement without significant washout.

Sedation with administration of thiopental and propofol during the procedure led to a paradoxical worsening of the movement disorder, accompanied by dysphoria and irritability. Adequate sedation was achieved with dexmedetomidine add-on.

Endoscopy showed mild congestive gastropathy with no evidence of esophageal varices.

Based on clinical, laboratory, and imaging findings, a diagnosis of chronic liver disease was made, characterized by normal liver and canalicular enzyme levels and preserved organ function.

### 3.3. Results of Laboratory Tests

From an immuno-hematologic standpoint, initial laboratory findings included hyporegenerative bilinear cytopenia (non-hemolytic anemia Hb 9 g/dL, MCV 75fL, reticulocytes 49,800/mmc, and thrombocytopenia PLT 99,000/mmc) and mild B-cell lymphopenia.

An occasional increase in serum lactic acid (5.3 mmol/L (0.5–2.2)) was detected.

### 3.4. Differential Diagnosis

One of the initial differential diagnoses considered was perinatal infection, particularly cytomegalovirus (CMV), which is characterized by microcephaly, intracranial calcifications, sensorineural hearing loss, chorioretinitis, growth restriction, hepatosplenomegaly, and jaundice.

After excluding all possible infectious causes (CMV, herpes, toxoplasmosis, rubella, HIV) through appropriate serological and molecular testing, we evaluated potential mitochondrial etiologies. Among the mitochondrial disorders considered were Sengers syndrome—caused by mutations in *AGK* or *SLC25A4* and characterized by congenital cataracts, hypertrophic cardiomyopathy, myopathy, and exercise-induced lactic acidosis—and Kearns–Sayre syndrome, a mitochondrial cytopathy presenting with ptosis, progressive external ophthalmoplegia, pigmentary retinopathy, cardiac conduction defects, ataxia, and hearing loss. Subsequently, we explored potential genetic etiologies, including Cockayne syndrome. This disorder is characterized by developmental delay and regression, progressive neurological decline, ataxia or movement disorders, microcephaly, sensorineural hearing loss, retinal involvement, growth failure, leukoencephalopathy, and multisystem involvement. Other genetic disorders considered in the differential diagnosis included Aicardi–Goutières syndrome, *COL4A1*-related disorders, Coats plus syndrome, Fahr disease, and Krabbe disease.

### 3.5. Genetic Tests and Final Diagnosis

Trio-based whole exome sequencing (WES) revealed the following compound heterozygous variants in the *KARS1* gene (NCBI RefSeq NM_001130089.2): c.617G>A p.(Arg206Gln) of paternal origin (MAF ~1/22,000 in East Asians, GnomAD 4.1) and c.1597C>A p.(Pro533Thr) of maternal origin (MAF 0, GnomAD 4.1). The child also carried two compound heterozygous *DNAH14* variants (NCBI RefSeq NM_001367479.1): c.7839 + 5G>A p.? of paternal origin, (MAF ~1:28,500 in Ashkenazi Jewish population and MAF 0 in other genetic ancestry groups, GnomAD 4.1) and c.12695C>T p.(Thr4232Ile) of maternal origin (MAF ~1:4000 in individuals of African descent, GnomAD 4.1).

Lastly, the heterozygous *PRKCG* variant c.1684G>T p.(Glu562*) (NCBI RefSeq NM_002739.5:) of paternal origin (MAF 0, GnomAD 4.1) was also found.

### 3.6. Possibile Precision Medicine Treatment

Based on in vitro and human studies reported in the literature [7,13,14] therapy with lysine monohydrate was initiated at the age of 8 months. However, due to a lack of a significant clinical and/or neuroradiological improvement, the treatment was discontinued after 7 months.

### 3.7. Follow-Up

The patient is undergoing a close multidisciplinary follow-up—namely neurological, ophthalmological, audiological, gastroenterological, immuno-hematological, and cardiological; he is also under weekly neurorehabilitation with physiotherapy and psychomotor therapy.

## 4. Narrative Literature Review

Using the previously defined criteria, we gathered 493 articles regarding KARS1. Studies addressing the involvement of KARS1 in cancer or tumorigenesis, or in other species were excluded from our review. Movement disorders have been described in 26 individuals with *KARS1*-related disorders, and their clinical features are summarized in Table S1 (Supplementary Materials). Among these patients, only seven cases exhibited hyperkinetic features, including: disorganized movements (1 case, [15]), chorea (1 case, [4]), bipyramidal syndrome combined with extrapyramidal movements (1 case, [13]), choreiform movements and myoclonus (1 case, [6]), extrapyramidal signs (1 case, [10]), abnormal movements (2 cases, [5]). In most cases, the age of onset of the movement disorder was not specified and video documentation is not available.

## 5. Discussion

Several variants of the *KARS1* gene reported in the literature have been associated with a wide and heterogeneous range of clinical phenotypes, including predominant neurological involvement with multisystem features consistent with infantile-onset progressive leukoencephalopathy with or without deafness (LEPID, OMIM # 619147) [4,10,15,16,17,18], congenital deafness and adult-onset progressive leukoencephalopathy (DEAPLE syndrome, OMIM #619196) [12,19], autosomal recessive sensorineural hearing loss (DFNB89, OMIM #613916) [20,21], and peripheral neuropathy resembling Charcot–Marie–Tooth disease type B (CMTB, OMIM #613641) [11].

The mechanisms underlying tissue-specific vulnerability in neurologic disorders linked to KARS remain unclear. Sun et al. proposed that dysfunction of cytoplasmic *KARS* is involved in peripheral neuropathy [5]. Conversely, most mitochondrial aminoacyl–transfer ribonucleic acid synthetase (ARS) defects primarily affect the central nervous system due to mitochondrial dysfunction [5].

LEPID represents the severe end of the clinical spectrum and is the most extensively characterized *KARS1* phenotype. Affected individuals present with an early onset global neurodevelopmental delay with a progressive course, leading to a severe neurocognitive impairment. Regression, often triggered by infections, has been reported [4,5,6,10,18]. Patients typically exhibit failure to thrive, congenital sensorineural hearing loss, microcephaly, hypotonia, motor impairment which may evolve into spastic tetraparesis, visual impairment, absent speech, and epilepsy. Systemic involvement has also been described, including hepatic, immuno-hematologic, cardiac, and renal manifestations [2,3,4,5,6,7,8,9,10]. Neuroimaging usually reveals abnormalities in deep white matter and cerebellar white matter, cortical atrophy, corticospinal tract involvement, and brain and spinal cord calcifications consistent with progressive leukoencephalopathy [4].

Our patient presented at age 5 months with a severe neurological phenotype, characterized by cervical–axial hypotonia, a hyperkinetic movement disorder, and orolingual dystonia with tongue protrusions.

Hyperkinetic movements are involuntary, excessive, and abnormal movements. They encompass a range of heterogeneous phenomena, such as chorea–ballism, dystonia, and other dyskinesias, which are characterized by distinct semiological manifestations, patterns, and sequences [22]. Dyskinesia refers to involuntary, repetitive, and sometimes jerky or writhing movements of the body, including the face, arms, legs, or trunk, and can vary in speed and intensity. Chorea consists of a continuous, random sequence of discrete involuntary movements that differ in timing, duration, direction, and anatomical location. Ballism is a form of chorea involving proximal joints (shoulder or hip), producing large-amplitude, flinging or flailing limb movements [22].

Specifically, in our patient, choreiform movements were observed in the axial and cervical regions; ballistic-type hyperkinesia predominated in the upper limbs, while frequent flexion–extension movements were noted in the lower limbs. Over the disease course (from 5 to 15 months of age), our patient experienced a mild improvement of his movement disorder.

This case broadens the phenotypic spectrum of *KARS1*-related disorders to include a prominent movement disorder with hyperkinetic features, which has not been previously described in detail.

Notably, movement disorders are often associated with metabolic diseases and have been described in conditions related to deficiencies in other aminoacyl-tRNA synthetases, including WARS2, CARS2, PARS2, RARS2, and AARS2 [23,24,25,26,27,28,29].

Movement disorders have also been increasingly recognized as a manifestation of childhood-onset mitochondrial diseases [30] although knowledge in infants remains limited. Interestingly, in the study by Ticci et al. [30], movement disorder was the presenting feature of mitochondrial diseases in 45/102 individuals, with a mean age at onset of 11 years. Ataxia was the most common movement disorder at onset, followed by dystonia, tremor, hypokinetic disorders, chorea, and myoclonus. During the disease course, most patients (67.7%) experienced worsening of their movement disorder. Basal ganglia involvement, cerebral white matter changes, and cerebellar atrophy were the most commonly associated neuroradiological patterns [30].

In our case, the movement disorder affects not only the limbs, trunk, and mouth but also the eyes. As regards ophthalmological findings described in the literature, random eye movements, nystagmus, and lack of fixation are the most commonly reported features [2,4,5,6,7,10,17,18]. The only available video documenting an ocular disorder in *KARS1*, by Cappuccio, shows a girl with intermittent strabismus, upward visual fixation, random eye movements, and lack of eye contact. Conversely, our patient presents with jerky eye movements, including a component of pendular horizontal nystagmus, accompanied by head movements. Furthermore, cases characterized by optic atrophy [2,6,10] and diffuse retinal abnormalities have been reported [2,10].

Our patient presents an apparently normal macular region and mild pigmentary irregularities at the posterior pole and mid-peripheral retina. Peluso described the diffuse and mottled retinal pigmentation, referring to it, for the first time, as a “leopard spot” retinopathy [2].

Our patient presented with bilateral sensorineural deafness and global developmental delay but has not experienced motor regression, even following a severe infection that progressed to sepsis at age 8 months. He exhibited congenital progressive microcephaly and failure to thrive in the first months of life, similarly to many of the individuals described in the literature [6,7,10].

Neuroimaging findings in our patient were predominantly characterized by cerebral and cerebellar calcifications, along with progressive severe atrophy and ventriculomegaly, both features frequently reported in individuals with KARS1 mutations [2,4,5,6,7,10,17,18,31]. In addition, a spinal MRI performed at 15 months of age revealed subtle T2 hypointense signal alterations, suggestive of calcifications, which have previously been reported in a minority of cases [4,10,16].

Cerebral atrophy [2,4,5,6,7,10,17,18], often associated with diffuse ventriculomegaly [4,6,7,18], has been consistently linked to *KARS1* mutations. Notably, in our patient, a rapid progression of cerebral atrophy with enlargement of the ventricles was documented. Similarly to previously described cases cerebral white matter abnormalities were observed [1,2,4,5,6,7,10,12,13,15,18,32]. Moreover, mild diffuse signal abnormalities were detected in the spinal cord.

The combination of cerebral and spinal calcifications with progressive cerebral atrophy and ventriculomegaly should be regarded as a key diagnostic indicator, raising suspicion for aminoacyl-tRNA-synthetase-related disorders, particularly those involving *KARS1* mutations.

Laboratory studies in many cases show increased serum lactate and deficiencies of mitochondrial respiratory chain complexes [2,4,7,9,10,13,15,33]; in our case a mild serum lactic acidosis has not been constantly detected.

The clinical phenotype of our patient—characterized by significant developmental delay, progressive microcephaly, movement disorder, and multiorgan involvement (including hepatic, hematologic, auditory, and ocular features), along with mild lactic acidosis and brain calcifications—raised clinical suspicion of a mitochondrial disorder, particularly a KARS1-related disorder, from the first evaluation. This diagnosis was subsequently confirmed by genetic testing.

Epilepsy, often drug-resistant, has been described in 37 individuals with *KARS1*-related syndrome so far, presenting with heterogeneous phenotypes including epileptic encephalopathies [2,5,6,7,10,13,17].

In our patient, subsequent EEGs since the first months of life enabled early identification of infraclinic seizures with a non-typical absence pattern triggered by IPS. Focal tonic asymmetric seizures appeared during follow-up occurring during both wakefulness and sleep.

In the literature, reported cases exhibit polymorphic seizures, and photosensitivity is not detailed.

Trio-based WES identified two compound heterozygous variants in *KARS1* (NM_001130089.2): the paternally inherited variant c.617G>A p.(Arg206Gln) and the maternally inherited variant c.1597C>A p.(Pro533Thr). Both missense variants are very rare, affect residues that are highly conserved across species and have not been previously reported in the literature in patients affected by *KARS1*-related disorders.

The Pro533 residue is located at the start of the α-helix within the catalytic domain, though it is far from the active site. As a result, variants in this region may affect the secondary structure of the catalytic domain, thereby altering the local conformation of the lysine binding domain and impacting the protein’s stability and function [5,7].

Two different variants at codon 533 have been reported as pathogenic in the literature, supported by functional validation analyses. Cappuccio et al. [7] described nine individuals with *KARS1* mutations, including two siblings carrying the p.(Pro533Arg) variant in compound heterozygosity with the p.(Ala57Pro) variant. In particular, a female with microcephaly, dysmorphic features, vertical gaze palsy in the first few months of life, nystagmus, and global developmental delay, who at 10 years developed epilepsy which was successfully treated with levetiracetam. Her brother showed global developmental delay, microcephaly, and vertical nystagmus, which later evolved into roving eye movements without tracking and unconjugated gaze. He developed infantile spasms, initially treated with ACTH and subsequently with vigabatrin and zonisamide [7]. Zhou et al. [12] and Sun et al. [5] described two siblings carrying the p.(Arg505His) and p.(Pro533Ser) variants in compound heterozygosity: a woman with congenital hearing loss and mild ID who developed progressive neurocognitive decline at age 25 (including ataxia and abnormal movements) and her brother, who also presented with congenital hearing loss [5,12].

Arg206 is located within the anticodon-binding domain and a pathogenic missense variant involving the adjacent residue has been reported in the literature. Cappuccio et al. described two affected siblings carrying the homozygous p.(Arg205Cys) variant, whose pathogenicity was confirmed by functional analyses [7].

According to a rigorous application of ACMG criteria, the two variants detected in our proband should be classified as variants of uncertain significance (VUS); however, considering the relatively specific clinical phenotype and the location of the mutated residues within the three-dimensional structure of the enzyme, it is reasonable to consider these variants as likely pathogenic [34].

The clinical relevance of the additional variants identified in *DNAH14* and *PRKCG* remains uncertain in our patient. Although *DNAH14* has recently been implicated in neurodevelopmental disorders, available data is limited. Splice site prediction tools indicate a potential splicing alteration associated with the c.7839+5G>A variant, whereas predictions regarding the p.(Thr4232Ile) missense change are inconclusive. Both variants are currently classified as variants of uncertain significance and further segregation analysis in additional offspring may help clarify their clinical significance.

The effect of truncating variants in *PRKCG* remains incompletely understood. While dominant Spinocerebellar Ataxia Type 14 (SCA14) is typically associated with missense mutations in *PRKCG*, truncating variants have been reported in homozygous or compound heterozygous states in patients with a recessive phenotype. Moreover, recent studies have described truncating variants in dominantly inherited forms that exhibit incomplete penetrance.

In general, segregation studies and functional assays play a critical role in the assessment of genetic variant pathogenicity, particularly in distinguishing variants of uncertain significance from those with disease-causing potential. Without such supporting evidence, there is a substantial risk of misclassifying rare or novel variants, potentially leading to inaccurate conclusions regarding their clinical relevance.

Many KARS1 pathogenic variants are believed to alter the local structure of the lysine-binding domain, resulting in decreased binding affinity for lysine. Lysine supplementation [7,13,14] has been shown to improve aminoacylation activity, thereby allowing it to meet high translational demand.

Based on the results of previous in vitro studies performed on yeast and the clinical improvement described by Bejma in one patient, a trial with lysine supplementation was administered. However, unfortunately, no significant benefits could be gained and the treatment was discontinued.

## 6. Conclusions

In conclusion, lysyl-tRNA synthetase 1 mutations pose a diagnostic challenge for clinicians due to their extreme phenotypic heterogeneity and variable expressivity. In this report, we describe a novel case with a prominent hyperkinetic movement disorder harboring biallelic *KARS1* variants, thereby contributing to expanding the phenotypic spectrum of *KARS1*-related disorders.

Our findings emphasize the importance of recognizing neurological manifestations, particularly movement disorders, associated with ophthalmological abnormalities (abnormal eye movements and retinopathy), hearing loss, and multisystemic involvement (hepatological, immuno-hematological) as essential diagnostic clues for this condition.

Additionally, the presence of brain and spinal cord calcifications combined with cerebral atrophy and ventriculomegaly should be regarded as key neuroradiological indicators.

All these clinical and radiological features should prompt consideration of the *KARS1*-related phenotypes in the differential diagnosis of complex neurogenetic syndromes and support the use of advanced genomic technologies to achieve faster and more accurate diagnoses.

## Supplementary Materials

The following supporting information can be downloaded at: https://www.mdpi.com/article/10.3390/neurolint17090143/s1, Video S1: Erratic eye movements and horizontal nystagmus bursts with a pendular component (age 8 months). Video S2: Subcontinuous hyperkinetic and choreo-ballistic movement disorders accompanied by head movements (age 8 months). Video S3: orolingual dystonia, grimacing and tongue protrusions (age 8 months). Table S1: Key clinical features of patients with movement disorders in KARS1-related disease.
