# Supplementary material for: Hyperkinetic Movement Disorder in KARS1-Related Disease: An Illustrative Video-Recorded Case and Narrative Literature Review
Source: Neurol Int. 2025 Sep 7;17(9):143. doi: 10.3390/neurolint17090143 (PMC12472568; doi:10.3390/neurolint17090143)
Supplement: Supplementary file 1 [file neurolint-17-00143-s001.zip › Table S1.pdf]

Table S1. Key clinical features of patients with movement disorders in Kars1-related disease.

Patient number (Gender)	Age at study report (Age at death)	Movement disorder	Developmental Delay	Regression	Microcephaly	Hearing loss	Nystagmus	Spasticity	Hypotonia	Epilepsy	Brain MRI changes	Authors
1 (M)	12y5m	Dystonia	+	+	NA	+	NA	+	-	+	+	Itoh [4]
2 (M)	3y5m	Chorea	+	+	NA	+	+	+	+	+	+	Itoh [4]
3 (M)	(4y)	Extrapyramidal movements	+	NA	NA	+	NA	+	+	+	+	Fuchs [13]
4 (F)	(20m)	Disorganized movements	+	NA	+	+	NA	NA	+	-	+	Ruzzenente [15]
5 (M)	7y	Extrapyramidal signs	+	+	+	+	+	+	+	+	+	Ardissone [10]
6 (M)	6y	Ataxia	+	+	-	+	+	+	-	+	+	Lin [6]
7 (F)	5y	Ataxia	NA	-	NA	+	NA	-	-	-	+	Lin [6]
8 (F)	3y	Ataxia	+	-	NA	+	NA	-	+	-	NA	Lin [6]
9 (F)	19y	Ataxia	+	-	+	+	NA	-	+	-	+	Lin [6]
10 (F)	55y	Ataxia, dystonia and tremor	+	-	NA	+	-	+	-	+	+	Lin [6]
11 (F)	20y	Ataxia	+	+	NA	+	-	-	+	+	-	Lin [6]
12 (M)	6y	Ataxia	+	-	+	+	NA	-	+	+	+	Lin [6]
13 (F)	(29y)	Choreiform movements and myoclonus	+	+	+	+	-	+	+	+	+	Lin [6]
14 (F)	26y	Ataxia and abnormal movements	-	-	NA	+	NA	+	+	NA	+	Sun [5]

Patient number (Gender)	Age at study report (Age at death)	Movement disorder	Developmental Delay	Regression	Microcephaly	Hearing loss	Nystagmus	Spasticity	Hypotonia	Epilepsy	Brain MRI changes	Authors
**15 (M)**	35y	Ataxia and abnormal movements	-	-	NA	+	NA	+	-	+	+	Sun [5]
**16 (F)**	11y	Ataxia and Bilateral hand tremor	+	+	NA	+	NA	+	+	+	+	Sun [5]
**17 (M)**	8y	Unspecified movement disorder	+	NA	+	-	-	NA	+	+	+	Cappuccio [7]
**18 (M)**	1y4m	Unspecified movement disorder	+	NA	+	+	+	+	NA	+	+	Cappuccio [7]
**19 (F)**	11y2m	Unspecified movement disorder	+	NA	+	-	+	NA	NA	+	-	Cappuccio [7]
**20 (M)**	2y1m	Unspecified movement disorder	+	NA	+	+	+	+	-	+	+	Cappuccio [7]
**21 (M)**	1y	Unspecified movement disorder	+	NA	+	-	+	+	-	+	+	Cappuccio [7]
**22 (M)**	17y4m	Unspecified movement disorder	+	+	NA	+	NA	NA	+	+	+	Cappuccio [7]
**23 (M)**	(3y)	Dystonia	+	NA	NA	+	NA	NA	+	NA	-	Lieber [33]
**24 (F)**	15y	Ataxia	+	NA	+	+	NA	NA	+	+ (abnormal EEG)	NA	Murray [31]

Patient number (Gender)	Age at study report (Age at death)	Movement disorder	Developmental Delay	Regression	Microcephaly	Hearing loss	Nystagmus	Spasticity	Hypotonia	Epilepsy	Brain MRI changes	Authors
**25 (F)**	(33y)	Ataxia, dystonia	+	-	NA	+	NA	NA	NA	NA	+	Scheidecker [32]
**26 (M)**	15m	Choreiform movement, ballistic-type hyperkinesia	+	-	+	+	+	-	+	+	+	Ferasin, (Present Study)

M=Male
F=Female

NA= Not Available
